# The Correlation Between Paternal Postpartum Anxiety and Maternal Postpartum Anxiety and Depression: A Cross‐Sectional Study

**DOI:** 10.1002/hsr2.72975

**Published:** 2026-08-05

**Authors:** Farzaneh Ismailzadeh, Parisa Samadi, Raziyeh Maasoumi, Shima Haghani, Fatemeh Alsadat Rahnemaei

**Affiliations:** ^1^ Department of Midwifery and Reproductive Health, School of Nursing and Midwifery Tehran University of Medical Sciences Tehran Iran; ^2^ Nursing and Midwifery Care Research Center, School of Nursing and Midwifery Tehran University of Medical Sciences Tehran Iran; ^3^ Nursing and Midwifery Care Research Center, Health Management Research Institute Iran University of Medical Sciences Tehran Iran; ^4^ Student Scientific Research Center (SSRC) Tehran University of Medical Sciences Tehran Iran

**Keywords:** anxiety, depression, psychological health, postpartum

## Abstract

**Background and Aims:**

Studies on paternal postpartum anxiety are sparse, and their results are mostly contradictory. Thus, this research was performed to determine the correlation between paternal postpartum anxiety and maternal postpartum anxiety and depression (PPD).

**Methods:**

This cross‐sectional study was conducted in 2023 among 250 couples who referred to selected health centers of Tehran University of Medical Sciences, using convenience sampling. The demographic questionnaire, short form of maternal postpartum specific anxiety and Edinburgh postpartum depression scale, and paternal Spielberger state‐trait anxiety scale were completed by phone and by the researcher. Data analysis was done using SPSS‐16 software, independent *t*‐tests, ANOVA, and correlation.

**Results:**

The paternal postpartum State and Trait anxiety were related with maternal postpartum anxiety (*r* = 0.250, *p* < 0.001) (*r* = 0.245, *p* < 0.001) and depression (*r* = 0.341, *p* < 0.001) (*r* = 0.416, *p* < 0.001), respectively.

**Conclusion:**

This research indicated that paternal postpartum anxiety is directly related to maternal postpartum anxiety and depression.

## 1 Introduction

The birth of a newborn is a major life transition for both mothers and fathers and is accompanied by substantial physical, hormonal, neurochemical, neurobiological, psychological, emotional, cognitive, and social changes. In addition to these individual adjustments, parents must renegotiate their identities and adapt to new familial roles. Together, these changes can increase vulnerability to psychological distress during the postnatal period [1, 2, 3]. Depression and anxiety are among the most common mental health concerns following childbirth and have been widely reported in both mothers and fathers [2, 4, 5, 6].

For fathers, the transition to parenthood presents specific challenges. The formation of a paternal identity, fears about fulfilling the parental role adequately, disruptions to daily routines, and responsibility for the family's financial security can all contribute to psychological strain [7, 8]. Reflecting these challenges, paternal anxiety is relatively common in the postnatal period, with prevalence rates of 25.4% at 3 months and 23.2% at 6 months after childbirth [3].

Paternal postpartum anxiety has been associated with adverse outcomes for fathers, mothers, and newborns. In fathers, it is linked to poorer psychological and physical health, reduced parenting confidence, strained social relationships, and lower quality of life. It may also increase stress, depressive symptoms, fatigue, family conflict, and functional impairment, while reducing perceived partner support. Importantly, anxiety in either parent can negatively affect the parent–child relationship and infant outcomes, including weight changes and emotional development. Evidence further suggests a direct association between paternal anxiety and anxiety symptoms in children [7, 9, 10].

A range of individual, familial, and socioeconomic factors have been identified as contributors to paternal postpartum anxiety. These include adverse childhood experiences such as neglect, exposure to harsh or violent parenting, physical, psychological, or sexual abuse, poor relationships with parents or peers during adolescence, limited parental support, early parental loss, and a history of anxiety in previous pregnancies. Socioeconomic factors, including lower educational attainment, lower income, maternal age and occupation, and work–family conflict, have also been associated with increased risk [7, 8, 10, 11, 12, 13, 14, 15].

Maternal mental health appears to play a central role in paternal postpartum anxiety. Previous studies have shown that maternal postpartum anxiety is a significant predictor of paternal anxiety during the postnatal period [10]. It is estimated that approximately 8%–12% of women in the United States experience postpartum anxiety each year [16]. Despite its prevalence, postpartum anxiety remains under‐recognized and under‐assessed. Maternal anxiety has been linked to impaired mother–infant interactions, disrupted feeding practices, altered mood and social functioning, and adverse neurological, cognitive, and emotional outcomes in infants [16, 17, 18, 19].

Maternal postpartum depression has also been identified as an important risk factor for paternal postpartum anxiety [10]. Globally, postpartum depression affects approximately 17.22% of women, with prevalence strongly associated with socioeconomic conditions and regional development [20]. This condition is associated with reduced breastfeeding, impaired mother–infant bonding, negative maternal mood, atypical neurological development, and emotional and behavioral problems in infants [16].

Overall, the evidence highlights the interdependence of maternal and paternal psychological health during the postnatal period. Psychological difficulties in one parent may increase the risk of mental health problems in the other, emphasizing the importance of perinatal care models that address parents' mental health both individually and as a couple [14, 21]. Despite its clinical relevance, postpartum anxiety—particularly in fathers—has received far less research attention than postpartum depression, and existing findings remain inconsistent [5, 7, 12]. Moreover, fathers generally have more limited access to postnatal mental health screening and intervention services, and significant barriers persist at both individual and healthcare system levels [13, 21]. As a result, knowledge about paternal psychological health remains limited, and optimal strategies for identifying and supporting fathers are not yet well established [21, 22, 23, 24].

Accordingly, the present study aimed to examine the association between paternal postpartum anxiety and maternal postpartum anxiety and depression.

## 2 Methods

### 2.1 Participants and Procedures

This cross‐sectional study took 4 months from June to September 2023.

According to the paper by Ierardi et al. [25], to determine the sample size with 95% confidence interval and with an estimation accuracy of *d* = 1 as well as an estimated standard deviation of 8.01, the minimum sample size required was an estimated 250 men together with their spouses.

The participants were selected using the available sampling method from couples referring to selected health centers, number 10, Farmanfarmayan, Shahid Ait and Akbar Abad (the largest population covered) to receive the health care of Tehran University of Medical Sciences. We used an available (convenience) sampling approach to recruit postpartum fathers and mothers attending primary health care centers for routine postnatal services. Recruitment took place between June and September 2023. All eligible parents who visited the centers during the data collection period were approached by trained research assistants and invited to participate.

Inclusion criteria were having Iranian nationality, having a child with an age range of 6 weeks to 1 year, having literacy of reading and writing, living with the spouse, having a term, healthy infant without complications, and the infant being a singleton. The exclusion criteria included a history of infertility, a history of referral to psychiatrists as well as taking psychiatric drugs (as self‐expressed by the person), a history of consuming drugs of abuse or psychotropic drugs (as self‐expressed by the person) as well a history of experiencing an event that would influence mood disorders over the past 6 months (as self‐expressed by the person).

After personal coordination and obtaining written informed consent, explanation: goals and methods, no compulsion to participate in the study, right to withdraw from the study, voluntary participation in the study, and assurance to the samples that their information and contact number will remain confidential, couples who agreed to participate in the study, due to the convenience of most parents and the fact that they do not have to spend a lot of time with their infant in healthcare centers, their phone numbers were taken and they were contacted at the right time, and the questionnaire was completed separately for men and with their spouses through a telephone interview. All data were collected through structured telephone interviews conducted by trained research assistants. Prior to data collection, interviewers completed a two‑session training workshop led by a clinical psychologist. The training included familiarization with the questionnaire content, instruction on standardized delivery of items, strategies for building rapport during phone communication, and guidelines for responding neutrally to participant questions. To ensure consistency, interviewers used a unified, pre‑scripted interview protocol that included standardized wording for all items and prompts. To minimize inattentive responding, interviewers were instructed to monitor for extremely rapid responses, repeated “don't know” answers, or inconsistencies between related items. When such patterns were detected, the interviewer paused, re‐read the relevant question, and confirmed the participant's intended answer. Cases with persistent inconsistencies or incomplete data were flagged for exclusion during data cleaning.

All questionnaires were checked for completeness immediately after each telephone interview. In cases where an item was skipped or unclear, the interviewer attempted to clarify the response during the same call. After data entry, the dataset was screened for missing values at both the item and scale levels.

Participants with more than 10% missing responses within a scale (e.g., the anxiety or depression subscales) were excluded from analysis following standard scoring guidelines. For cases with only one or two missing items within a validated scale, mean substitution was applied based on the participant's own valid responses within that scale, consistent with commonly recommended procedures for psychological instruments. No multiple imputation methods were used due to the low proportion of missing values and the cross‐sectional nature of the data. Sensitivity checks comparing analyses with and without substituted values showed no meaningful differences in the results.

### 2.2 Measures

#### 2.2.1 Demographic Checklist

Characteristics such as age, education, job, insurance, exercise, sleep, alcohol consumption, smoking, whether their wife's pregnancy was desired or not, frequency of pregnancy, and number of infants were assessed.

#### 2.2.2 Spielberger State‐Trait Anxiety Inventory (STAI)

To assess paternal postpartum anxiety, the Spielberger state‐trait anxiety inventory was utilized. This scale is a 40‐item self‐report instrument that measures the situational and general anxiety of the person (by determining the State and Trait anxiety of the person). It includes two 20‐item scales presenting separate state and trait anxiety criteria [26, 27]. State anxiety means anxiety is in the form of a transient emotion where general states of nervosity, frustration, and worry are evaluated with self‐report criteria. Trait anxiety refers to the tendency to perceive different situations as threatening and stressful and thus experience high levels of state anxiety. The cutoff point for STAI (40 or more) is chosen [5]. The validity and reliability of STAI were investigated and confirmed in 1973 by Spielberger and in Iran in 2018 in Hemti Maslakpak's study, which had a high‐reliability coefficient of 0.8 [28]. The Persian version of the scale has been prepared, and its reliability has also been confirmed [26].

#### 2.2.3 Postpartum Specific Anxiety Scale‐Among Iranian Women (PSAS‐IR)

The PSAS‐IR scale was used to measure maternal postpartum anxiety. This scale comprises four subscales: maternal competence and attachment anxiety, concerns about infant safety and welfare, anxiety related to infant care performance, and psychosocial adaptation to motherhood [17]. Given the large number of items in the original version, the 16‐item version has been identified as the most theoretically and psychometrically robust form of the scale.

The reliability of the scale has been confirmed using internal consistency (Cronbach's alpha) and test–retest reliability methods. The validity of the four‐factor structure has also been supported by satisfactory goodness‐of‐fit indices.

The 16‐item Postpartum Specific Anxiety Scale – Research Short Form (PSAS‐RSF) has demonstrated strong psychometric properties and reliability. The PSAS‐RSF is the first validated short form specifically developed to measure postpartum anxiety (PPA). Findings indicate that the scale is theoretically sound, statistically robust, reliable, and valid, and it can be used up to 12 months postpartum.

Each factor consists of four items. Responses are rated on a four‐point Likert scale: never, sometimes, often, and almost always. The response options are scored from 1 to 4, respectively. The total possible score ranges from 16 to 64 [29, 30].

Regarding validity assessment, face validity indicated that all items were clear, unambiguous, and appropriately difficult, with the lowest impact score being 1.5. In the content validity assessment, all items achieved the minimum acceptable levels of the Content Validity Ratio (CVR) and Content Validity Index (CVI). The overall CVR and CVI values for the scale were 0.88 and 0.89, respectively.

The internal consistency reliability of the scale, as measured by Cronbach's alpha, was 0.93, indicating strong internal consistency. Test–retest reliability, assessed using the Intraclass Correlation Coefficient (ICC), was 0.92. Construct validity further confirmed the adequacy of the scale [17].

#### 2.2.4 Edinburgh Postnatal Depression Scale (EPDS)

The Edinburgh depression scale was employed for measuring the postpartum depression of mothers. It is a self‐report scale developed by Cox et al. It includes 10 items. EPDS is utilized for postpartum depression screening and expresses the severity of depression symptoms over the past 7 days. EPDS has satisfactory specificity and sensitivity and is also sensitive to changes in the degree of depression over time. It is also a simple scoring method, where each item has four possible responses ranging from 0 to 3, with a minimum score of zero and a maximum score of 30 [31, 32]. In this research, to assess maternal postpartum depression, the acquisition of a score of 13 and higher from EPDS over 6 weeks up to 1 year postnatally has been considered [33, 34]. Holden et al. reported that this questionnaire has good validity, and using Cronbach's α, its reliability is higher than 0.80. Ahmadi et al. reported a reliability of 0.70 among the Iranian population [35].

### 2.3 Ethical Considerations

For ethical considerations, the research protocol was approved by the ethics committee for medical research of Tehran University of Medical Sciences (code of ethics: IR.TUMS.FNM.REC.1401.182, date: 18.02.2023). In a face‐to‐face arrangement, the researcher explained the objectives of the study to the subjects and assured them of the confidentiality of their data. Then, the phone numbers of the couples who agreed to participate in the research were taken, and they were contacted at the right time. Also, in the first question of the questionnaire, they were asked if they were satisfied with participating in the research.

### 2.4 Statistical Analysis

Demographic data were reported by descriptive statistics, including frequency, mean, and standard deviation and assessment of the correlation, ANOVA, independent *t‐*tests, and the Pearson correlation coefficient were used. *p* < 0.05 was considered significant. Data analysis was performed by SPSS 16.

## 3 Results

The mean age of fathers and mothers in the study was 36.18 ± 5.928 years and 31.756 ± 5.809 years, respectively (Table 1). Most mothers had high education (58.8%) and were housewives (77.6%).

**Table 1:** Frequency distribution of personal characteristics and their relationship with the state and trait anxiety of fathers.

Personal characteristics of the father		Frequency (percent)	State anxiety		Test result	Trait anxiety		Test result	Test type
			Mean	SD		Mean	SD		
Age (years)	< 30	26 (10.4%)	40.615	11.906	*r* = 0.001 *p* = 0.989	38.884	12.384	*r* = 0.001 *p* = 0.989	Correlation test
	30–39	160 (64%)	35.837	10.384		34.612	9.73		
	40–49	56 (22.4%)	37.0	9.129		36.642	9.688		
	≥ 50	8 (3.2%)	44.0	17.121		37.375	13.276		
Education	High school	33 (13.2%)	39.878	11.813	*F* = 2.237 *p* = 0.109	38.181	10.616	*F* = 2.237 *p* = 0.109	ANOVA test
	Diploma	81 (32.4%)	37.407	10.834		36.469	10.72		
	University	136 (54.4%)	35.794	10.158		34.455	9.64		
Job	Non‐employee	97 (38.8%)	37.907	10.394	*t* = 1.243 df = 248 *p* = 0.215	37.907	10.394	*t* = 1.243 df = 248 *p* = 0.215	Independent *t*‐test
	Employee	153 (61.2%)	36.189	10.797		36.189	10.797		
Insurance	Has it	238 (95.2%)	36.31	10.415	*t* = −3.692 df = 248 *p* < 0.001	36.31	10.415	*t* = −3.692 df = 248 *p* < 0.001	Independent *t*‐test
	Does not have	12 (4.8%)	47.666	9.93		47.666	9.93		
Exercise per week	No	123 (49.2%)	39.065	11.056	*F* = 5.429 *p* = 0.005	36.837	10.007	*F* = 1.84 *p* = 0.161	ANOVA test
	Yes (once or twice)	55 (22%)	35.018	9.084		34.69	8.726		
	Yes (more than twice)	72(28.8%)	34.486	10.419		34.18	11.32		
Sleep disorder	Yes	58 (23.2%)	40.431	10.483	*t* = −2.961 df = 248 *p* = 0.003	40.431	10.483	*t* = −2.961 df = 248 *p* = 0.003	Independent *t*‐test
	No	192 (76.8%)	35.776	10.495		35.776	10.495		
Alcohol consumption	Yes	29 (11.6%)	39.586	10.22	t = −1.471 df = 248 *p* = 0.370	39.586	10.22	*t* = −1.471 df = 248 *p* = 0.143	Independent *t*‐test
	No	221 (88.4%)	36.497	10.681		36.497	10.681		
Smoking	Yes	60 (24%)	37.933	11.144	*t* = 0.898 df = 248 *p* = 0.370	37.933	11.144	*t* = 0.898 df = 248 *p* = 0.370	Independent *t*‐test
	No	190 (76%)	36.515	10.503		36.515	10.503		
Desired pregnancy	Yes	215 (86%)	36.358	10.657	*t* = −1.84 df = 248 *p* = 0.067	36.358	10.657	*t* = −1.84 df = 248 *p* = 0.067	Independent *t*‐test
	No	35 (14%)	39.914	10.262		39.914	10.262		
Gravida	First birth	109 (43.6%)	36.559	11.062	*F* = 0.82 *p* = 0.442	35.367	10.819	*F* = 0.755 *p* = 0.471	ANOVA test
	Second birth	84 (33.6%)	37.988	10.517		36.619	10.032		
	Third birth and more	57 (22.8%)	35.754	10.075		34.543	9.116		
Child	First child	125 (50%)	36.947	10.95	*F* = 0.016 *p* = 0.984	36.453	10.882	*F* = 1.499 *p* = 0.225	ANOVA test
	Second child	101 (40.4%)	36.333	7.094		31.333	6.11		
	Third child and more	24 (9.6%)	36.726	10.343		34.368	8.956		

The means of state anxiety and trait anxiety were calculated as 36.856 ± 10.655 and 35.6 ± 10.18, respectively. The mean scores of maternal postpartum anxiety in the research were obtained at 27.832 ± 7.875. The mean score of the maternal postpartum depression was obtained at 8.988 ± 5.136.

The paternal postpartum state anxiety had a direct correlation with maternal postpartum anxiety (*p *< 0.001, *r *= 0.25) and depression (*p *< 0.001, *r *= 0.341)(Table 2) as well as the paternal postpartum trait anxiety had a direct correlation with maternal postpartum anxiety (*p *< 0.001, *r *= 0.245) and depression (*p *< 0.001, *r *= 0.416) (Table 3).

**Table 2:** Association of paternal postpartum anxiety with maternal postpartum anxiety and depression.

Variable	Paternal anxiety	
	State	Trait
Maternal anxiety	*r* = 0.25	*r* = 0.245
	*p* < 0.001	*p* < 0.001
Maternal depression	*r *= 0.341	*r* = 0.416
	*p* < 0.001	*p* < 0.001

**Table 3:** Results of linear regression of factors affecting state anxiety in fathers.

Factors affecting state anxiety		Coefficient	Standard coefficient	Test statistics	Significant level	Confidence interval	*R* ^2^
Insurance status	Yes	−8.855	−0.178	−3.045	0.003	(−14.583, −3.127)	0.194
	Does not have	Reference					
Exercise	Yes, once or twice a week	−3.880	−0.151	−2.448	0.015	(−7.002, −0.758)	
	Yes, more than twice a week	−2.669	−0.114	−1.845	0.066	(−5.518, 0.180)	
	No	Reference					
Sleep disorder	No	−3.214	−0.128	−2.178	0.030	(−6.120, −0.308)	
	Yes	Reference					
Postpartum depression in mothers		0.594	0.286	4.601	0.00	(0.340, 0.849)	
Postpartum anxiety in mothers		0.117	0.087	1.358	0.176	(−0.053, 0.287)	

To assess the factors associated with fathers' state and trait anxiety, multiple linear regression analysis was performed using the enter method. Variables that were statistically significant in the univariate analyses were included in the multivariable regression model. Categorical variables were converted into indicator variables prior to analysis.

The normality of residuals was confirmed. Multicollinearity among independent variables was assessed using the variance inflation factor (VIF), and all VIF values were below 2, indicating no multicollinearity concerns. The independence of errors was examined using the Durbin–Watson test, yielding values of 1.882 for the state anxiety model and 1.778 for the trait anxiety model. These values indicate that the assumption of independence of errors was satisfied. Therefore, the regression models were considered appropriate for the analysis. The multiple linear regression model showed that having insurance, exercising once or twice a week, sleep disturbance, and maternal postpartum depression were significant, such that the model coefficient indicated that state anxiety in fathers who had insurance was 8.855 units lower than in fathers who did not have insurance People who exercised once or twice a week had a 3.880 lower level of state anxiety than people who did not exercise, and people who did not have sleep disorders had a 3.214 lower level of state anxiety than people who did have sleep disorders. Also, with an increase in postpartum depression in the spouse, state anxiety increased by 0.594. Also, the standard coefficient of the model indicated that maternal postpartum depression had the greatest relationship with state anxiety, with a standard coefficient of 0.286. The coefficient of determination of the model was 0.194. In other words, the significant variables in the model are 0.194 related to the dependent variable, that is, state anxiety (Table 3). The multiple linear regression model showed that having insurance, not having a sleep disorder, and postpartum depression in the mother were significant, so that the model coefficient indicated that trait anxiety in fathers who had insurance was 8.116 units lower than in fathers who did not have insurance. In those who did not have a sleep disorder, the level of trait anxiety was 4.926 units lower than in those who had a sleep disorder. Also, with the increase in maternal postpartum depression, trait anxiety increases by 0.751 units, and the standard coefficient of the model indicates that maternal postpartum depression had the greatest relationship on trait anxiety, with a standard coefficient of 0.379. The coefficient of determination of the model was 0.236. In other words, the significant variables in the model are 0.236 related to the dependent variable, namely, trait anxiety (Table 4).

**Table 4:** Results of linear regression of factors affecting trait anxiety in fathers.

Factors affecting trait anxiety		Coefficient	Standard coefficient	Test statistics	Significant level	Confidence interval	*R* ^2^
Insurance status	Yes	−8.116	−0.171	−3.027	0.003	(−13.397, −2.836)	0.236
	Does not have	Reference					
Sleep disorder	No	−4.928	−0.205	−3.653	0.00	(−7.585, −2.271)	
	Yes	Reference					
Postpartum depression in mothers		0.751	0.379	6.289	0.00	(0.516, 0.986)	
Postpartum anxiety in mothers		0.043	0.033	0.537	0.592	(−0.115, 0.200)	

## 4 Discussion

The findings of the present study indicated that fathers' State and Trait anxiety were significantly and positively correlated with maternal postpartum anxiety. This result is consistent with previous evidence suggesting that maternal anxiety during the postnatal period plays a substantial role in paternal psychological well‐being. A systematic review conducted by Philpott et al. [10] identified maternal postpartum anxiety as an important predictor of paternal anxiety following childbirth. Similarly, Ierardi et al. [25], in a study examining depression and anxiety in mothers and fathers and their association with mother–infant interactions at 3 months postpartum among 70 couples, reported findings aligned with the present study using comparable assessment tools for paternal anxiety and maternal depression. In addition, Henshaw et al. investigated psychosocial factors related to symptoms of depression and early anxiety after childbirth in primiparous women and their partners and found that maternal anxiety was a significant factor associated with paternal anxiety during the postpartum period, despite using different instruments to assess anxiety [36].

In addition to maternal anxiety, the present study demonstrated that paternal State and Trait anxiety were significantly and positively associated with maternal postpartum depression. In other words, higher levels of maternal postpartum depression were accompanied by higher levels of anxiety in fathers. This finding is consistent with previous research. Ierardi et al. [25] reported a significant correlation between maternal postpartum depression and paternal State and Trait anxiety. Moreover, a systematic review by Philpott et al. [10], as well as a longitudinal follow‐up study by Vismara et al. [3] examining the consequences of stress, anxiety, and perinatal depression in first‐time mothers and fathers from 3 to 6 months postpartum, similarly showed that maternal depression was associated with increased paternal anxiety. These converging findings support the notion that maternal depressive symptoms may act as an important stressor influencing paternal emotional regulation during the postnatal period.

Taken together, the results of the present study reinforce the interconnected nature of maternal and paternal psychological health following childbirth. The observed associations suggest that psychological difficulties in mothers, including anxiety and depression, may increase vulnerability to anxiety in fathers. Therefore, addressing the psychological well‐being of fathers alongside mothers during pregnancy and the postnatal period should be considered a critical public health priority. Integrating paternal mental health screening and support into perinatal care may contribute to improved outcomes for parents and the family system as a whole.

## 5 Strengths and Limitations

While this study has strengths, such as the use of accurate data collection tools, a relatively large sample size, and data collection up to the first year after delivery [33], it suffers from some limitations as well. This study has several limitations that should be acknowledged. First, the use of available (convenience) sampling may limit the generalizability of the findings. Parents who attend primary health centers and agree to participate may differ from those who do not seek routine postnatal care, which introduces the potential for selection bias.

Second, all measures relied on self‐report instruments, which are subject to recall bias, social desirability bias, and potential under‐ or over‐reporting of emotional symptoms. Although standardized and validated tools were used, self‐report assessments cannot fully capture clinical diagnoses.

Third, data collection was conducted via telephone interviews. While this method improves accessibility and reduces participant burden, it also limits the interviewer's ability to observe nonverbal cues, may reduce participant engagement, and may influence the accuracy of responses in sensitive topics. Despite using a standardized script and quality‐control procedures, telephone‐based assessments inherently carry constraints compared to face‐to‐face interviews.

Finally, the cross‐sectional design prevents any inference of causality between paternal and maternal postpartum psychological symptoms. Longitudinal research is recommended to better understand temporal relationships.

## 6 Conclusion

The findings of this cross‐sectional study showed that there is a positive correlation between maternal postpartum depression and anxiety and paternal postpartum anxiety. The findings of the present study indicated that it is essential for healthcare providers to provide fathers with appropriate services and training during this period. However, appropriate policymaking is necessary first. Then, its implementation and evaluation should be carried out by healthcare providers. It is hoped that fathers' mental health programs will be designed and included in safe maternal programs.

## Author Contributions

**Farzaneh Ismailzadeh:** conceptualization, investigation, writing – original draft. **Parisa Samadi:** methodology, validation, visualization, writing – review and editing, supervision. **Raziyeh Maasoumi:** data curation, supervision, visualization. **Shima Haghani:** software, formal analysis. **Fatemeh Alsadat Rahnemaei:** writing – review and editing, resources.

## Funding

The authors have nothing to report.

## Conflicts of Interest

The authors declare no conflicts of interest.

## Transparency Statement

Dr. Parisa Samadi affirms that this manuscript is an honest, accurate, and transparent account of the study being reported; that no important aspects of the study have been omitted; and that any discrepancies from the study as planned (and, if relevant, registered) have been explained.

## Data Availability

Data sharing does not apply to this article as no new data were created or analyzed in this study.
